# Digital platforms and entrepreneurial support: a field experiment in online mentoring

**DOI:** 10.1007/s11187-022-00704-8

**Published:** 2022-10-25

**Authors:** Saurabh A. Lall, Li-Wei Chen, Dyana P. Mason

**Affiliations:** 1 Adam Smith Business School, University of Glasgow, Glasgow, UK; 2 Strome College of Business, Old Dominion University, Norfolk, USA; 3 School of Planning, Public Policy and Management, University of Oregon, Eugene, USA

**Keywords:** Entrepreneurship, Mentoring, Entrepreneur, Mentee, Online, Digital entrepreneurship

## Abstract

The benefits of entrepreneurial mentorship are well documented, but there is limited research on how entrepreneurs connect with mentors, especially in digital settings. We partnered with an online platform that connects entrepreneurs to potential mentors to conduct a field experiment in online mentoring. Drawing on literature on entrepreneurial mentorship and Social Cognitive Theory, we compared the effects of three interventions on the likelihood of reaching out and making a connection with a mentor in a digital setting. We find that showing entrepreneurs a video of a successful mentor–mentee relationship increases the chances that they will reach out to a potential mentor but does not improve their chances of making a connection. These findings are more pronounced for female entrepreneurs. While not all entrepreneurs adopt the offered interventions, those that make the effort to learn to navigate the online platform and craft a suitable introductory message are successful in establishing a mentoring connection. We discuss these implications for both theory and practice.

## Abstract (plain language summary)

Showing entrepreneurs an example of a successful mentor–mentee relationship makes them more likely to reach out to a potential mentor, and even more so for women entrepreneurs. Additionally, entrepreneurs that make the effort to learn to navigate an online mentoring platform and craft a suitable introductory message are successful in establishing a mentoring connection. We partnered with a global online platform to test three interventions to encourage entrepreneurs to reach out to mentors. However, since many entrepreneurs did not take up the offered interventions, the real-world implications of these approaches are less positive, and overall result in few connections with mentors. We suggest that requiring entrepreneurs to undertake some basic training before engaging on online platforms may increase the barrier to entry but can ultimately lead to more meaningful connections with mentors.

## Introduction

“While everyone says that you should have a mentor, no one provides details on how.” – Richard Branson (Founder and CEO, Virgin Group).

Mentoring relationships are a conduit through which individuals gain tangible and intangible resources. Mentorship is known to offer several benefits to entrepreneurs, including increasing the propensity to pick entrepreneurial career paths (Eesley & Wang, 2017), improving entrepreneurial learning (Schou et al., 2021), reducing fear of failure (Nanda & Sørensen, 2010), increasing self-confidence (St-Jean, 2011), and increasing opportunity recognition (Ozgen & Baron, 2007). Entrepreneurs seek advice from a variety of sources including professional business consultants, local chambers of commerce, and networking groups, among others (Kuhn et al., 2017). There are diminishing returns to seeking advice from extremely large networks (Semrau & Werner, 2014; Watson, 2007), and a supportive mentor can help entrepreneurs with more targeted and relevant advice. However, despite the positive perception of mentorship, there has been relatively little attention paid to examining the antecedents of acquiring a mentor — particularly in online or digital environments. Moreover, while past studies have examined mentoring dyads and the evolving relationships between entrepreneurs and their mentors, few studies have addressed the more fundamental issue of how entrepreneurs can find a mentor in the first place.

Entrepreneurs value advisors but need to identify quality sources that are most beneficial for their own particular goals and situations (Kuhn et al., 2017). Reaching out to a stranger for help may not come naturally to all entrepreneurs, and there are a range of potential inhibiting factors. For instance, women entrepreneurs are inhibited by the systemic lack of resources (Brush et al., 2019), suitable training options (Davis & Abdiyeva, 2012), and insufficient social networks (Neumeyer et al., 2019), and have to rely more on friends and family (Robinson & Stubberud, 2009). Furthermore, entrepreneurial mentoring has often been assumed to be primarily in-person and highly localized (Bennett et al., 2000; Kuhn et al., 2017). With the emergence of online platforms and social media which allow entrepreneurs to access a wider pool of potential mentors, many of the obstacles that prevent traditionally underserved entrepreneurs from finding mentors should be overcome (Kuhn & Galloway, 2015; Kuhn et al., 2017). In this study, we seek to answer two research questions — how can we encourage entrepreneurs to reach out to a potential mentor in an online setting, and consequently, does this outreach result in an ongoing mentoring relationship?

Thus, our study on online mentoring tackles significant questions that have not yet been answered. Online mentoring offers the opportunity of mentor–mentee tie formation through informal, spontaneous relationships instead of highly structured programs that assign mentors to entrepreneurs. Studies of tie formations have been conducted on online matching platforms in diverse phenomena like online dating (Bapna et al., 2016; Hitsch et al., 2010), career guidance (Vaast, 2021), and crowdfunding in entrepreneurship (Allison et al., 2015; Anglin et al., 2019; Mollick, 2014). Yet, the literature on ties formation in online mentoring is under-developed, and to our knowledge, there have been no causal studies testing theories of tie formation in an online entrepreneurial mentoring context.

We use a field experimental^1^ design with real entrepreneurs and mentors active on an online platform to determine if specific interventions can support an entrepreneur’s effort to connect with a mentor. While randomized field experiments have been widely used to study entrepreneurship programs in development economics (see McKenzie, 2020 for a meta-analysis), they are a relatively recent import to the broader field of entrepreneurship and innovation (Hsu et al., 2017; Williams et al., 2019), and offer several benefits over lab experiments (Huizingh & Mulder, 2015). Partnering with a large international nonprofit organization that operates an online mentoring platform for entrepreneurs (referred to as the platform provider), we develop three interventions that are rooted in Social Cognitive Theory (Bandura, 1977) and qualitative insights. These interventions test ways to help entrepreneurs understand *what* to expect from a mentoring relationship, *how* to reach out to a potential mentor, and to become more proficient at navigating the online setting. Our findings provide lessons specifically for online mentoring programs, but also more broadly for mentoring.

## Literature review

### Entrepreneurial networks and mentorship

Social capital and social networks are critical in the launch of new ventures, the performance of established businesses, and overall firm growth (Kuhn et al., 2017; Stam et al., 2014). While there is extensive research on how and why entrepreneurs create new ties (Vissa, 2012), much of the attention on entrepreneurial networks tends to focus on relationships that involve some form of economic exchange with customers, investors, or suppliers (van RijnSoever, 2020; Vissa, 2011, 2012). Mentorship, in contrast, is a form of entrepreneurial support that is typically motivated by the principle of “paying it forward,” or helping someone because one has been helped in the past (Kay & Wallace, 2009), and has recently gained attention in entrepreneurship as a critical component of entrepreneurial ecosystems (Alaassar et al., 2022; Brown & Mason, 2017; Belitski & Büyükbalci, 2021). Mentors are typically successful entrepreneurs or business professionals who provide advice and connect new entrepreneurs to those in their network (Brown & Mason, 2017). Geographically connected networks result in useful knowledge spillovers for small, entrepreneurial firms (Audretsch et al., 2022), access to resources, knowledge of how to use those resources, and a greater understanding of the entrepreneurial ecosystem in which they are based (Belitski and Büyükbalci, 2021; Cao & Shi, 2021). Ecosystem perspectives are increasingly relevant as policymakers and scholars examine the determinants of successful regional growth (Audretsch & Belitski, 2017, 2021; Audretsch et al., 2021). Thus, studying the antecedents of informal networks (Belitski and Büyükbalci, 2021) is an important avenue for research on ecosystems.

Although mentors provide valuable human and social capital for entrepreneurs, forming a mentor–mentee relationship is challenging for nascent entrepreneurs. While mentoring relationships may emerge over shared identities and interests, it can be intimidating for a new entrepreneur to reach out and ask for advice when they are unsure of their capabilities or their business (Dimitriadis & Koning, 2020; Radu Lefebvre & Redien-Collot, 2013). Kalbfleisch and Eckley (2003) note that it is largely the role of the mentee or protégé to initiate the relationship. In most formal entrepreneurial support programs, mentors and mentees are recruited and matched by program staff (St. Jean, 2011). If entrepreneurs are not able to access entrepreneurial support programs in the first place, they have little means to form a mentor–mentee relationship in a face-to-face context.

#### ***Digitalcommunities of support for entrepreneurs***

Much of the literature to date has focused on a geographical access to entrepreneurial resources and support. Increasingly, entrepreneurs are seeking support through more informal channels such as online communities and forums (Schou et al., 2021). Online forums and digital mentorship have gained even more importance since 2020, offering entrepreneurs critical social support during the COVID-19 pandemic (Meurer et al., 2022; Giones et al., 2020). Reflecting a new paradigm of digital entrepreneurial ecosystem (Nambisan, 2017; Sussan & Acs, 2017; Song, 2019), digital mentorship platforms and forums afford entrepreneurs opportunities to access and engage with and learn from a broader and more diverse set of actors than they may have traditionally had access to in their communities (Schou et al., 2021). Meanwhile, scholars emphasize the need to focus on the intermingling of the digital and human side of interactions in online settings (Garud & Giuliani, 2013; Nambisan, 2017; Schou et al., 2021). While digital infrastructure may lead to improved access to resources (Aldrich, 2014), we still do not know if it will work effectively for entrepreneurs who traditionally lack access to mentors. Developing trust and high-quality interpersonal relationships can be challenging in computer-mediated environments (Lewandowski et al., 2011; Rockmann & Northcraft, 2008; Wilson, et al., 2006), and these issues may be further compounded by entrepreneurs’ education (Lee & Jones, 2008), gender, and age (Kuhn et al., 2017). Furthermore, digital mentorship may replicate existing inequities for traditionally marginalized entrepreneurs (e.g., women) (Neumeyer et al., 2019).

Recently, scholars have started to understand how entrepreneurs participate in online communities (Schou et al., 2021) which offers new digital opportunities for entrepreneurial learning that disregard boundaries of time, geographical distance, and hierarchy (Hwang et al., 2015; Nambisan, 2017; Autio et al., 2013). Here, it is helpful to distinguish between more open online communities where entrepreneurs may post questions to their peers in an anonymized manner (e.g., Reddit) and more structured online communities with relatively stable membership that require entrepreneurs and mentors to provide identifying information about themselves and describe their businesses and experience. Past research suggests that open forums may lead to more fleeting connections, without a sense of membership and engagement (Vaast, 2021) while more structured communities can contribute to a deeper and more involved relationship (Vaast, 2021; Meurer et al., 2022).

Establishing a more engaged relationship with a mentor is more costly and daunting for entrepreneurs than engaging with an anonymous online community of peers. Therefore, we implement our study in the latter type of setting, where we can test interventions that result in the successful initiation and establishment of these mentor–mentee dyadic relationships by entrepreneurs. In the following section, we combine lessons from scholarship in social and cognitive behavior (Bandura, 1977) with qualitative insights from entrepreneurs on the platform to identify three types of barriers: First, entrepreneurs may lack understanding of *what* benefits they can gain from mentorship. Second, they may lack the skills on *how* they can reach out to a mentor. Finally, it is possible they are simply struggling to navigate the online setting.

### Conceptual framework and hypotheses

Social Cognitive Theory (SCT) (Bandura, 1977, 1986) offers practical guidance on how people can be encouraged to develop certain competencies in online settings. SCT is a psychosocial model that suggests that behavior, cognition, and environmental factors can all influence each other to drive personal change and development, and has been widely used in education, and entrepreneurial education specifically (Austin & Nauta, 2016; Segal et al., 2007; Zozimo et al., 2017). Three mechanisms of this theory are particularly relevant for entrepreneurial learning — vicarious learning (using role models), enactive mastery (generating small wins through practice), and verbal persuasion (Rideout & Gray, 2013). Each of these may provide incentives, or constraints, to an entrepreneur who seeks a mentor.

#### Vicarious learning to explain the value of mentorship

Entrepreneurs seek and receive advice from a variety of sources such as friends and family, paid advisors like lawyers and accountants, and local chambers of commerce (Kuhn & Galloway, 2015; Kuhn et al., 2017), and as such all of these actors can be considered part of an entrepreneurial ecosystem that can nurture and support entrepreneurs and their ventures (Belitski and Büyükbalci, 2021) As their networks grow, there are diminishing returns to seeking advice — making it difficult to assess what these different relationships can offer (Semrau & Warner, 2014). In addition, entrepreneurs are more likely to engage into high growth activities if they perceive that relevant knowledge and expertise may be available to them in their environment (Audretsch et al., 2021). However, the value of the information and support provided by mentorship is difficult to judge, since it is typically voluntary in nature, and does not include any economic exchange (Mason et al., 2021). Additionally, during initial interactions, there is often uncertainty about what is expected of each party (Brashers, 2001), and lack of knowledge about the potential value of the relationship (Dahlander & McFarland, 2013). Entrepreneurs are increasingly more comfortable seeking advice from peers in the form of coactive vicarious learning (Schou et al., 2021), but the more traditional form of mentor–mentee relationship is fraught with inhibitions, especially on the mentee side (Radu Lefebvre & Redien-Collot, 2013). While entrepreneurs may have a vague idea of the benefits of mentoring, they are unlikely to understand exactly how to reach out for support, and what to expect in a new mentoring relationship (Radu Lefebvre & Redien-Collot, 2013), and this uncertainty can inhibit them from reaching out (Engel et al., 2017). This uncertainty is likely to be exacerbated for women entrepreneurs, who may have a lower degree of social capital than male entrepreneurs (Neumeyer et al., 2019). We therefore hypothesize that observing an example of a successful mentor–mentee relationship is likely to help entrepreneurs appreciate the value of a mentor and better understand *what* to expect from a mentoring relationship.

- Hypothesis 1a: Promoting vicarious learning by providing entrepreneurs with a vivid example of a mentor-mentee relationship will lead to a higher likelihood of them initiating contact with a potential mentor.
- Hypothesis 1b: Promoting vicarious learning by providing entrepreneurs with a vivid example of a mentor-mentee relationship will lead to a higher likelihood of establishing a mentoring connection.

#### Enactive mastery to overcome lack of confidence

Entrepreneurs have traditionally been viewed as confident individuals (Miller, 2015), but many entrepreneurs struggle with asking for outside help at the early stages of starting their business (Dimitriadis & Koning, 2020), mostly relying on friends (Bennett & Chatterji, 2019). While the growth of online communities offers new avenues for support (Schou et al., 2021), reaching out to a mentor on an online platform may seem daunting to many entrepreneurs. Establishing a new connection can be costly, in terms of time, effort, and patience (Dimitriadis & Koning, 2020), and entrepreneurs may lack the confidence to approach a potential mentor. This lack of confidence is even more pronounced for entrepreneurs that are traditionally marginalized, such as women entrepreneurs (Brush et al., 2019). Scholars have pointed out the importance of framing questions on online communities and how that can result in different ways that advice is offered (Meurer et al., 2022). Thus, it is essential for entrepreneurs to gain some experience in *how* they approach a potential mentor.

Our second intervention relies on the principle of enactive mastery (Bandura, 1977), or the idea of building “small wins” for entrepreneurs that may be hesitant to reach out to a mentor. Past research suggests accomplishing small-scale, low-stakes tasks can improve entrepreneurs’ confidence and self-efficacy (Rideout & Gray, 2013; Segal et al., 2007). Providing hands-on practice in connecting with a mentor may enable them to reach out to mentors on their own.

- Hypothesis 2a: Promoting enactive mastery by simulating a connection with a mentor will help entrepreneurs gain more confidence and lead to a higher likelihood of them initiating contact with a potential mentor.
- Hypothesis 2b: Promoting enactive mastery by simulating a connection with a mentor will help entrepreneurs gain more confidence and lead to a higher likelihood of establishing a mentoring connection.

#### Verbal persuasion to help entrepreneurs use the platform

Finally, we recognize that some of the factors hindering entrepreneurs from connecting with mentors may relate to entrepreneurs being overwhelmed by the platform, and not expecting to make a successful connection. The issue of “platform literacy” — learning to navigate the platform (Waldkirch et al., 2021, p.2659) is critical to finding success in a range of digital settings. Navigating networks effectively enables entrepreneurs to not only access relevant resources, but also provides them with the knowledge to use these resources effectively (Belitski and Büyükbalci, 2021; Elfring & Hulsink, 2003; Srećković, 2018). Access to information and the ability to use information technology effectively has also been linked to venture success by helping connect entrepreneurs to needed resources and networks (Audretsch & Belitski, 2017). Online platforms can offer a range of options — entrepreneurs on online crowdfunding platforms (e.g., Kickstarter, Kiva) and marketplaces (e.g., Etsy) need to learn to effectively navigate the platform to their advantage and attract potential customers and investors (Allison et al., 2015; Mollick, 2014; Waldkirch et al., 2021). Understanding how to explain their businesses and describe the help they need effectively in an online setting may seem like a staggering task for an overburdened entrepreneur. Thus, our third hypotheses (and related intervention) focus on the idea of building platform literacy.

- Hypothesis 3a: Encouraging entrepreneurs to make the most of the platform and providing them with tips on what has worked for others will lead to a higher likelihood of them initiating contact with a potential mentor.
- Hypothesis 3b: Encouraging entrepreneurs to make the most of the platform and providing them with tips on what has worked for others will lead to a higher likelihood of establishing a mentoring connection.

## Data and methods

To test these hypotheses, we conducted a randomized, controlled field experiment in partnership with a nonprofit organization. While experimental methods are increasingly popular in entrepreneurship research (see for example, Eesley & Wang, 2017; Huizingh & Mulder, 2015) laboratory or classroom settings involving students or online survey participants (e.g., Amazon Mechanical Turk) are most common (Hsu et al., 2017). Field experiments offer some significant advantages over lab experiments in terms of generalizability and participation bias (Al-Ubaydli & List, 2013), but provide researchers with less fine-grained control of the environment in which the experiment is conducted, require higher costs, and often have unforeseen implementation challenges (Czibor et al., 2019). However, they have the important advantage of demonstrating how certain interventions may play out under “real world” conditions, which is critical for generalizing to entrepreneurship policy and practice (Huizingh & Mulder, 2015). In our study, we are able to test these interventions with real entrepreneurs and mentors, effectively demonstrating whether these interventions could be translated into practice.^2^

The nonprofit we work with in this study (which we call the *platform provider*) provides a free online mentoring platform in English, Spanish, and French for entrepreneurs from around the world seeking support from mentors. Entrepreneurs register on the site by completing an online profile that describes their venture and their needs. The platform was initially launched in 2001 and has undergone several iterations. It has been in operation in its current form since 2015 and has over 47,000 registered entrepreneurs and 18,000 mentors. The platform shows up on the first page of Google search results when searching for the terms “business mentor” and “entrepreneur mentor,” which suggests that it is accessible to a wider range of entrepreneurs than more targeted programs. The platform is open to entrepreneurs from a variety of industries and forms, including social enterprises and both for-profit and nonprofit organizations. The platform provider provides additional guidance to all entrepreneurs on how to reach out to potential mentors, and how to design an attractive profile on the site.

Unlike most entrepreneur mentorship programs, however, those that join the platform are not matched formally to a mentor. Instead, they are provided with a list of mentors suggested by an algorithm that matches users based on interests, industry, location, and expertise. Entrepreneurs are free to reach out to any potential mentors they feel may best meet their needs (see Appendix 1 figs. 2, 3, and 4 for a visual description of the platform). Mentors also signed up on the platform are also free to reach out to entrepreneurs directly. Once an entrepreneur reaches out to potential mentors, the mentors have the option to reply, or not. The open nature of the platform makes it a suitable setting for a field experiment on establishing a mentoring relationship since all participants can be assumed to be interested in finding a mentor or receiving mentor-style information and services but are free to decide with whom they want to engage.

### Experimental design

We tested the effects of three interventions on the likelihood of entrepreneurs reaching out to a potential mentor, and eventually building a mentoring relationship. Importantly, working with real entrepreneurs and mentors means we did not face the generalizability challenges of lab experiments using online workers or students (Czibor et al., 2019). Since we are interested in the real-world effectiveness of these interventions, we offered interventions that would be typical choices provided by the platform provider. Working with a field partner, rather than in a laboratory setting, we also account for practical considerations of cost, effort, and feasibility when developing the three interventions. Our interventions took place among entrepreneurs who created new profiles on the platform between May and August 2019 in either English or Spanish.^3^ The final trial includes 3583 entrepreneurs. Sample sizes and power for each intervention and the control group were calculated using extant historical data and expectations derived from our research questions. We found that the trial would require a minimum of *N* = 443 per group to generate 95% confidence at 0.80 power, for a total of 1772 entrepreneurs (Gelman & Hill, 2007) over the course of the experiment. To be conservative, we aimed for a considerably higher (more than double) sample size in our experiment, resulting in 3583 observations. After removing 561 observations due to missing gender information, we are left with a final sample size of 3022. The chi-square test on the equality of proportions of missing data across groups indicates that there is no statistically significant difference on the distribution of missing values across groups (${\chi }^{2}$ = 1.578, *p* = 0.664).

**Fig. 1:**
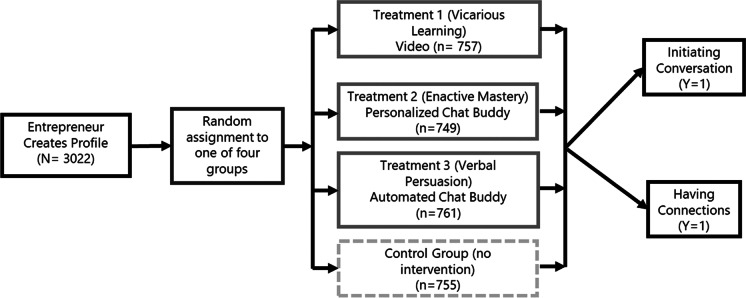
Experimental design with randomized groups

**Fig. 2:**
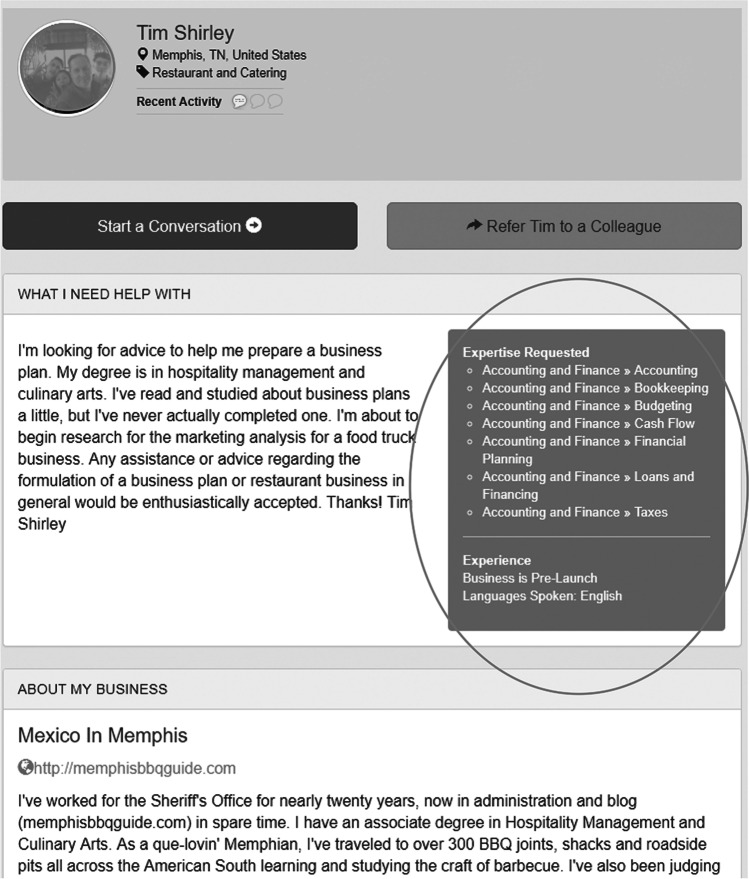
Entrepreneur creates a profile on the platform and states what they need help with. *Entrepreneurs in the treatment groups are offered the treatment after completing the profile*

**Fig. 3:**
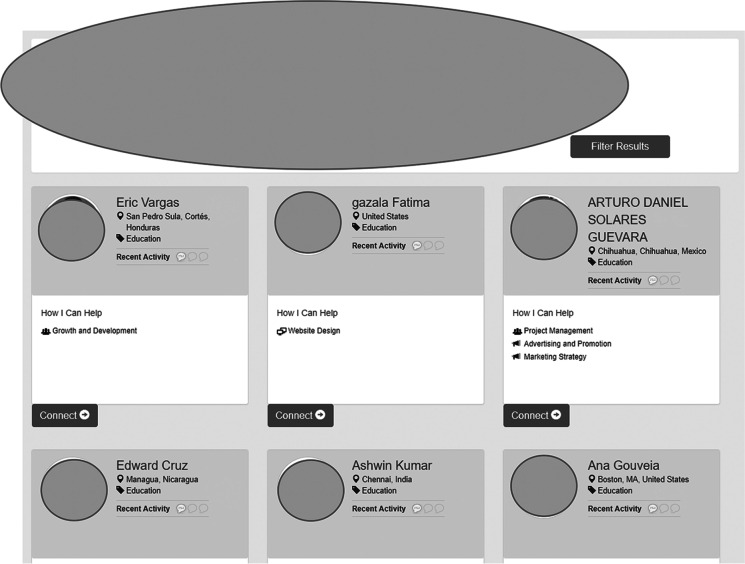
Entrepreneur sees potential mentor matches based on their needs

**Fig. 4:**
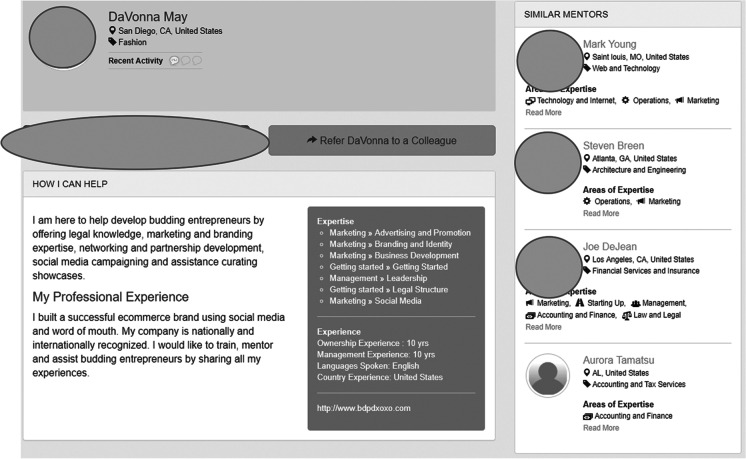
Entrepreneur can review mentor profiles and reach out to a mentor

**Fig. 5:**
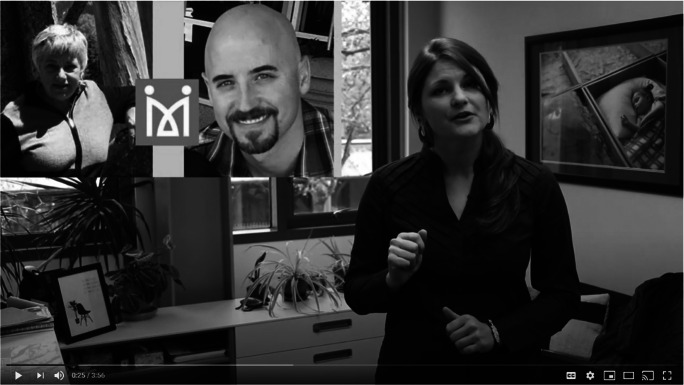
Screenshot of Video Message

Once entrepreneurs signed up and created a new profile, they were sequentially assigned to one of three treatment groups or a control group. Each entrepreneur received only one treatment (intervention), or none (control group). All entrepreneurs maintained the same access to all platform services and functions, such as messaging mentors for consultation. In this study, we use conversation data between May and November 2019 so that entrepreneurs have at least 3 months to establish connections.^4^ In addition to the literature, we reviewed historical data on the platform to better understand the characteristics of entrepreneurs that successfully connected with mentors. Finally, we reviewed some historical qualitative feedback from entrepreneurs and mentors on the platform to understand factors that might be preventing a possible connection. Three recurring themes that emerged on the entrepreneur side were (a) lack of a clear understanding of what a mentor can offer^5^; (b) lack of confidence in reaching out to a potential mentor^6^; and (c) difficulty in understanding the platform and how to use it effectively. We drew on the literature in Social Cognitive Theory (Bandura, 1986) and these practical insights to develop the interventions,^7^each of which emphasized a solution to tackle one of these issues. All three interventions (described in Appendix 2 Fig. 5) encouraged entrepreneurs to be proactive and reach out to potential mentors. The experimental groups, which can also be seen in Fig. 1, are:

1. Treatment Group 1 (*Vicarious Learning*): To test Hypothesis 1a and 1b, entrepreneurs received an invitation to view an online video about the benefits of online mentoring. We worked with the platform provider to develop a professionally produced video that depicted a vivid example of a successful mentoring relationship between Bonnie (the mentor) and Travis (the mentee). The 4-min video^8^ focused on describing how Bonnie helped Travis grow his business, clearly illustrating the value of a good mentoring relationship. The video closed by encouraging entrepreneurs to reach out to a prospective mentor and mentioning several resources they could find on the platform. Based on the principle of Vicarious Learning (role modelling), the video primarily focuses on showing an example of a successful mentor–mentee relationship on the platform (Bonnie and Travis).
2. Treatment Group 2 (*Enactive Mastery*): To test Hypothesis 2a and 2b, a second group received a personalized “chatbot” message. We worked with the platform provider to develop a personalized “chatbot” module that would be displayed on the screen for new registrants and walk them through the process of connecting with a mentor — by providing sample text for a first message and simulating a connection with a mentor. Once the simulated connection was made, the “chatbot” encouraged the entrepreneur to reach out to an actual prospective mentor and mentioned other resources they could find on the platform. The message was sent by a real person^9^ and focuses on how to reach out to a mentor, including a sample message. Based on the mechanism of enactive mastery (generating small wins), this treatment was designed to help improve the confidence of entrepreneurs by simulating a connection with a mentor. This intervention is delivered by an employee of the platform provider, who provides personalized support to an entrepreneur in the form of a sample introductory message, along with a simulated connection to a mentor.
3. Treatment Group 3 (*Verbal Persuasion*): Finally, to test Hypothesis 3a and 3b, entrepreneurs received an automated “chatbot” message that informs the user about effective strategies on using the platform, and how to provide the type of information mentors are looking for (Verbal Persuasion). We also developed an automated “chatbot” script that emphasized the platform’s ease-of-use and explained how it could help entrepreneurs connect with a mentor who could help them grow their business. This treatment is designed to help entrepreneurs navigate the platform and alleviate potential barriers related to the structure of the online setting. These prompts are delivered by an automated “chatbot” through the platform and aim to persuade entrepreneurs to reach out to a mentor.
4. Control Group: These individuals do not receive any intervention but have access to all standard platform benefits and information.

Our treatments reach the participants consistently across groups. We used Google Analytics to keep track of the views of the vicarious learning video (Treatment 1). Our sample totaled 3022 participants who were assigned to one of the three treatment groups, or the control group. Among the 916 participants assigned to Treatment 1, 605 unique watching events are recorded, and 282 incidents reach 50% of the video or higher. We have 885 participants assigned to Treatments 2 and 3 each. In the Enactive Mastery group (Treatment 2), 418 participants read the message; among them, 161 participants responded. Similarly, we have 415 participants read the message and 144 responded among those who read the message. We do not find statistical difference in the proportions of participants who actively responded to the treatment, suggesting that our treatments reach the participants consistently. After removing observations with missing data, we have 353 and 368 participants in Treatment 2 and Treatment 3 respectively for the treatment-on-treated analysis.

### Variables

#### Dependent variables

We have two dependent variables in this study. *Initiating Conversations* is a binary variable coded as 1 if an entrepreneur initiates at least one conversation with a potential mentor on the platform within 3–6 months^10^ and 0 otherwise. *Having Connections* is the second dependent variable, coded as 1 if an entrepreneur establishes at least one meaningful connection with a mentor and 0 otherwise. A meaningful connection is defined as four or more messages exchanged between an entrepreneur and a mentor in one conversation. While we recognize that this variable does not capture the quality or depth of a mentoring relationship, an exchange of this nature provides an indication of a back-and-forth conversation with a mentor and is also used by the platform provider as a suitable proxy for a conversation. We conduct robustness checks with two alternative connection variables in the following section.

#### Control variables

We include three control variables in our models, based on self-reported entrepreneur profile data. The variable *Sex* captures the entrepreneur’s self-reported gender and is coded as 1 if the entrepreneur is a female and 0 if male. *Age* captures the entrepreneur’s age in years when at the time of creating the profile. Finally, *Native Language (Spanish)* is coded as 1 if an entrepreneur’s native language is Spanish and 0 if it is English.

## Analysis

The summary statistics with the correlation matrix are presented in Table 1. Thirty-nine percent of the 3022 participants have initiated at least one conversation with a mentor, but only 14% have developed a meaningful connection. Roughly one-third (31%) of the entrepreneurs are female, and the main age of the entrepreneurs is 34 years. Finally, about a quarter (23%) use Spanish as their native language.

**Table 1:** Summary statistics and correlation matrix (*N* = 3022)

	Mean	SD	Med	Min	Max	1	2	3	4	5
1. Initiating Conversation (Yes = 1)	0.39	0.49	0.00	0.00	1.00	1.00				
2. Having Connections (Yes = 1)	0.14	0.35	0.00	0.00	1.00	0.44	1.00			
3. Sex (Female = 1)	0.31	0.46	0.00	0.00	1.00	− 0.01	0.04	1.00		
4. Age	34.04	7.23	34.06	18.00	85.00	− 0.06	− 0.01	− 0.02	1.00	
5. Native Language (Spanish)	0.23	0.42	0.00	0.00	1.00	− 0.11	− 0.05	0.05	0.11	1.00

We further validate if the randomization has been implemented properly by calculating the means by groups for each variable in Table 2. The sample size for each group is roughly the same at about 750. One-way ANOVA tests show that the group means are not statistically different for sex (*F* = 0.038, *p*-value = 0.99) and age (*F* = 1.391, *p*-value = 0.244). We do find an unequal distribution of Spanish-speaking participants among the groups (*F* = 2.425, *p*-value = 0.0638), which may be due to the late introduction of the Spanish treatment.

**Table 2:** Group means by experimental groups

Experimental group	*N*	Gender	Age	Initiating Conversation	Having Connection	Native Language (Spanish)
Control	755	0.31	33.83	0.37	0.14	0.21
Treatment 1: Vicarious Learning	757	0.31	34.20	0.41	0.15	0.27
Treatment 2: Enactive Mastery	749	0.32	33.74	0.40	0.15	0.22
Treatment 3: Verbal Persuasion	761	0.31	34.40	0.37	0.12	0.24

Based on our pre-registration, we report intent-to-treat (ITT) estimates for our analyses. The ITT estimates compare groups based on random assignment but do not account for compliance. For field experiments, this estimate is more relevant because it allows practitioners and policy makers to determine what the effectiveness of treatment would look like in the real world (Angrist & Pischke, 2014), since not all entrepreneurs will take up the offered support. We conduct two logistic regression models to examine the effect of the treatments on the entrepreneurs’ ability to connect with a mentor. The model for subject $i$ under treatment $j$ is specified as follows.

$$\mathrm{Probability}(\mathrm{Initiating}\hspace{0.25em}\mathrm{Conversations}\hspace{0.25em}\mathrm{or}\hspace{0.25em}\mathrm{Having}\hspace{0.25em}\mathrm{Connections})\;=\alpha+\beta_jx_{ij}+\gamma_i\mathrm{Control}s_i$$

The estimated models are presented in Table 3. From column 1, we see that being a female, being older, or having Spanish as the native language negatively affected the chance of entrepreneurs reaching out to a mentor. The effects of sex and age are not statistically significant in the model regarding making a connection (column 2). However, Spanish-speaking entrepreneurs are still less likely to have meaningful connections ($\beta$ = − 0.847, *p*-value = 0.020) than English-speaking entrepreneurs.

**Table 3:** Logistic regression models of treatment effects on obtaining mentorship (intent-to-treatment)

	*Dependent variable*	
	Initiating Conversation (Yes = 1)	Having Connections (Yes = 1)
	(1)	(2)
Treatment 1: Vicarious Learning	0.229**	0.163
	(0.110)	(0.151)
Treatment 2: Enactive Mastery	0.127	0.114
	(0.111)	(0.152)
Treatment 3: Verbal Persuasion	0.012	− 0.112
	(0.111)	(0.157)
Sex (Female = 1)	− 0.190**	0.011
	(0.088)	(0.119)
Age	− 0.011**	− 0.001
	(0.006)	(0.007)
Native Language (Spanish)	− 0.847**	− 0.870*
	(0.365)	(0.450)
Constant	1.021**	− 0.459
	(0.513)	(0.642)
Country effects?	Yes	Yes
Observations	3022	3022
Log likelihood	− 1901.051	− 1155.152
Akaike Inf. Crit	3984.102	2492.303

^*^p < 0.1; **p < 0.05; ***p < 0.01

We then turn our attention to the effect of treatments, with mixed results. Treatment 1 (*Vicarious Learning*), which involves watching a video with a vivid example of a mentoring relationship, has a statistically significant positive effect on entrepreneurs’ likelihood to initiate conversations with mentors ($\beta$ = 0.229, *p*-value = 0.038), supporting Hypothesis 1a. In other words, the chance of initiating conversations with mentors is increased by 26% if entrepreneurs watch the video. We do not find a statistically significant effect of *Vicarious Learning* on making a successful connection ($\beta$ = 0.163, *p*-value = 0.283) while the sign of the coefficient is consistent with our prediction, finding no support for Hypothesis 1b. We do not see the same results from either Treatment 2 (*Enactive Mastery*) or Treatment 3 (*Verbal Persuasion*). Therefore, Hypotheses 2 (a and b) and 3 (a and b) are not supported under the ITT setting. In sum, our intent-to-treat analyses find that vicarious learning (role modelling) is effective in encouraging entrepreneurs to reach out to potential mentors, but unfortunately insufficient in convincing mentors to respond.

Given the historical challenges in accessing mentorship faced by female entrepreneurs, we further examine the subsample of female entrepreneurs on the platform. We conduct the same analyses on the subsample of female entrepreneurs in Table 4 and observe a similar pattern. None of the treatments is effective in improving the chance of building a meaningful connection with mentors. However, the effect of Treatment 1 (*Vicarious Learning*) on initiating a conversation is statistically significant and positive ($\beta$ = 0.452, *p*-value = 0.026), and the effect size is considerably larger. The chance of a female entrepreneur reaching out to a mentor is increased by 57% after she watches the video.^11^

**Table 4:** Logistic regression models of treatment effects on obtaining mentorship (female entrepreneurs, ITT)

	*Dependent variable*	
	Initiating Conversation (Yes = 1)	Having Connections (Yes = 1)
	(1)	(2)
Treatment 1: Vicarious Learning	0.452**	0.197
	(0.203)	(0.274)
Treatment 2: Enactive Mastery	0.247	0.258
	(0.202)	(0.268)
Treatment 3: Verbal Persuasion	0.046	0.025
	(0.207)	(0.279)
Age	0.016	0.015
	(0.010)	(0.013)
Native Language (Spanish)	− 1.166	− 0.617
	(0.682)	(0.762)
Constant	0.567	− 0.922
	(0.888)	(1.011)
Country effects?	Yes	Yes
Observations	946	946
Log likelihood	− 573.761	− 372.280
Akaike Inf. Crit	1265.522	862.559

^*^p < 0.1; **p < 0.05; ***p < 0.01

These findings represent the lower bounds of effectiveness of our strategies and provide an important real-world summary of our results. In subsequent robustness checks, we account for compliance in two treatments, which enables us to also understand effectiveness when only observing compliers.

### Robustness checks

The online mentoring platform allows us to examine whether a message sent is read by the recipient. Therefore, we can conduct a treatment-on-treated (ToT) analysis for Treatments 2 and 3.^12^ Table 5 reports the results based on the treatment-on-treated principle. The sample size of Treatments 2 and 3 is restricted to those who read the message sent from the nonprofit organization employee or the chatbot, resulting in 353 and 368 observations each. The sample size is unchanged for Treatment 1 and the control group, resulting in 2233 observations in total.

**Table 5:** Logistic regression models of treatment effects on obtaining mentorship (treatment-on-treated)

	*Dependent variable*	
	Initiating Conversation (Yes = 1)	Having Connections (Yes = 1)
	(1)	(2)
Treatment 1: Vicarious Learning	0.233**	0.152
	(0.110)	(0.151)
Treatment 2: Enactive Mastery	0.711***	0.697***
	(0.138)	(0.170)
Treatment 3: Verbal Persuasion	0.828***	0.806***
	(0.136)	(0.165)
Sex (Female = 1)	− 0.231**	0.011
	(0.101)	(0.127)
Age	− 0.011*	− 0.0001
	(0.006)	(0.008)
Native Language (Spanish)	− 0.960**	− 1.015*
	(0.461)	(0.529)
Constant	1.263**	− 0.217
	(0.619)	(0.713)
Country effects?	Yes	Yes
Observations	2233	2233
Log likelihood	− 1416.656	− 973.533
Akaike Inf. Crit	2999.313	2113.067

^*^p < 0.1; **p < 0.05; ***p < 0.01

The treatment-on-treated analysis show that both Treatment 2 (*Enactive Mastery*) and Treatment 3 (*Verbal Persuasion*) have positive statistically significant effect on initiating conversations ($\beta$ = 0.711, *p*-value = 0.000 and $\beta$ = 0.828, *p*-value = 0.000, respectively). In addition, both treatments show positive effect on building connections with mentors ($\beta$ = 0.697, *p*-value = 0.000 and $\beta$ = 0.806, *p*-value = 0.000, respectively). This result suggests that if the participants are actually treated, namely reading the message sent, both treatments contribute to building connections with mentors. However, in the real world, people may not attend to treatments even when they receive one, and these treatments may not achieve their intended results due to lack of uptake. The randomization of the original samples helps to ensure that unobservable characteristics of the participants are equally distributed among the treatments and control.^13^ Nonetheless, there may still be some residual selection effects within the treatment group in this treatment-on-treated analysis.

We return to the ITT setting and conduct a further series of robustness checks to verify our findings (Table 6). First, it is possible that the statistically insignificant results of social capital development may be due to insufficient time for connections to develop. We re-construct the *Having Connections* variable one year after the experiment. However, we still do not find any intent-to-treat effect on developing connections with mentors compared to the control group.

**Table 6:** Generalized linear models of treatment effects on obtaining mentorship (ITT)

	*Dependent variable*		
	Having Connections (1 year later)	Having Mentor Contact Info	Number of Days Taken to Initiate First Conversation
	(1)	(2)	(3)
Treatment 1: Vicarious Learning	0.147	0.075	− 0.061
	(0.150)	(0.134)	(0.275)
Treatment 2: Enactive Mastery	0.190	− 0.070	0.048
	(0.149)	(0.137)	(0.282)
Treatment 3: Verbal Persuasion	0.024	− 0.199	− 0.033
	(0.152)	(0.139)	(0.281)
Sex (Female = 1)	− 0.062	0.420^***^	− 0.040
	(0.116)	(0.104)	(0.224)
Age	− 0.002	0.0004	− 0.022
	(0.007)	(0.007)	(0.224)
Native Language (Spanish)	− 0.622	− 0.961^**^	2.520^***^
	(0.444)	(0.447)	(0.948)
Constant	− 0.567	0.284	2.077
	(0.627)	(0.601)	(1.281)
Country fixed effect	Yes	Yes	Yes
Observations	3,022	3,022	945
Log likelihood	− 1,207.485	− 1,365.645	− 36,450.860
Akaike Inf. Crit	2,596.971	2,913.290	73,037.710

^*^p < 0.1; **p < 0.05; ***p < 0.01

Second, it is possible that conversations occur off the platform because mentors may have their preferred means of communication. We therefore construct another dependent variable in the second model in Table 6. Instead of using the number of messages exchanged as the indicator of building a connection, we note whether a mentor provides the advice-seeking entrepreneur their private contact information as an indicator of building a connection. Our partner nonprofit organization uses text analysis techniques to identify potential private contact information, such as emails, phone numbers, Skype, and so on. The variable *Having Mentor Contact Info* is coded as 1 if any of the conversation involves the exchange of mentor’s private contact information and 0 otherwise. The results are consistent with our existing findings.

Finally, we want to verify if our treatments motivate the entrepreneurs to reach out to mentors (model 3 in Table 6). If the treatment is effective, we should expect entrepreneurs to reach out to a mentor sooner than those in the control group. *Number of Days Taken to Initiate First Conversation* measures the number of days taken by the entrepreneurs to initiate their first conversation with a mentor. On average, it takes the control group 21.5 days to initiate the first conversation, comparing with 19.8 days, 21.2 days, and 25 days from Treatments 1, 2, and 3. Entrepreneurs who took the *Vicarious Learning* treatment initiated their conversations earlier than the control group, consistent with our previous finding. Since the distribution of the days shows a pattern of overdispersion, we use a negative binomial model to analyze this data. The third column of Table 6 shows that while the coefficients of Treatments 1 and 3 are negative, the standard deviations are too large to make them statistically significant.

## Discussion and conclusion

This study experimentally tested theories of behavior that may support tie formation between entrepreneurs and mentors in a geographically dispersed digital environment. In doing so, we contribute to the literature in two important ways. First, our findings suggest that while some interventions may have an effect in incentivizing entrepreneurs in seeking support from a mentor online, establishing a mentor–mentee relationship remains difficult on digital platforms and warrants further study. While online communities of support offer considerable promise, actually “nudging” entrepreneurs to pro-actively seek assistance for venture development is still challenging. Second, we provide the methodological contribution of using a randomized controlled field experiment to measure the effect of different interventions on entrepreneur behavior. Our findings highlight that the effectiveness of online entrepreneurial support platforms may need more effort to be fully realized. While online mentoring offers unique benefits, particularly by offering increased accessibility to those who do not typically have access to in-person mentoring opportunities, the challenges of making a meaningful connection in a virtual space may continue to be difficult for many. In addition, as the growth of online entrepreneurial support has gained new importance during the pandemic, our field experiment offers important insights on how mentoring relationships may be initiated and established in a relatively fluid digital setting — distinct from formal programs where one is assigned a mentor (Brooks et al., 2018; Lall et al., 2020), or the informal relationships entrepreneurs establish within their communities and ecosystems (Belitski and Büyükbalci, 2021; Brown & Mason, 2017; Radu Lefebvre & Redien-Collot, 2013).

Developing new relationships can be a costly and daunting task (Dimitriadis & Koning, 2020; Stam et al., 2014) and requires active effort on the part of the entrepreneur, especially in digital settings (Schou et al., 2021). Since it is largely up to the mentee or protégé to initiate the relationship (Kalbfleisch & Eckley, 2003), our findings offer several important practical implications. First, we note that the very idea of mentoring is not one that is always easy to grasp — mentors occupy a liminal space, falling between a paid consultant and a friend (Radu Lefebvre & Redien-Collot, 2013). Entrepreneurs may not understand *what* to expect from a mentoring relationship, and *how* to approach a mentor online, preventing them from reaching out.

Second, we find that uncertainty about the value of mentorship can be overcome through vicarious learning (showing role models). Viewing a video that describes an example of a successful mentoring relationship is effective in encouraging entrepreneurs to reach out to potential mentors. This treatment is particularly effective for female entrepreneurs, who have historically been less likely to reach out to mentors on the platform (consistent with past research on gender and entrepreneurial mentorship — for instance, see Orser et al., 2012). We speculate that since the mentor in the example is a woman, and the video is also presented by a female staff member of the platform provider, female entrepreneurs are more convinced of the value of mentoring and motivated to reach out, based on the principle of homophily (McPherson et al., 2001).^14^

Our other two approaches, based on enactive mastery (by simulating a connection and generating a “small win”) and verbal persuasion (to improve platform literacy), are not effective in our intent-to-treat estimates, and in fact none of our treatments results in an ongoing connection with a mentor — illustrating the challenges of establishing meaningful relationships in online environments. However, once we account for non-compliance, the enactive mastery and verbal persuasion approaches are more effective, and ultimately even lead to establishing a mentoring connection for entrepreneurs that take up the treatment. These supplementary analyses suggest a promising avenue for the possible need to require some initial training when entrepreneurs initially sign up on digital platforms.

We note several limitations of our study. First, we do not know if entrepreneurs and mentors move their conversations off the platform after contact information is exchanged, which would not be uncommon. While we can examine the exchange of certain types of contact information such as email addresses or phone numbers, we are unable to track this activity beyond that point. Second, the platform lacks detailed demographic information on the socio-economic status of entrepreneurs and mentors, and only allows us to control for gender, age, and language. Third, we are only able to monitor compliance for two of the three interventions, so unable to determine how effective the vicarious learning treatment is on entrepreneurs that comply (which is likely to be even higher than the ITT estimates). Fourth, we were only able to test entrepreneur-focused interventions in this study; we are still unable to determine exactly what leads to the dyadic mentor–mentee relationship. Fifth, while our interventions were differentiated for the entrepreneurs, it is possible that they did not offer enough difference to drive a meaningful outcome in our dependent variables.

Future research would benefit from testing additional theories of human behavior that may be relevant to tie formation and the development of networks and resulting social capital, especially in an entrepreneurial ecosystem context (Audretsch & Belitski, 2017). These may include providing information or asking entrepreneurs to complete a simple task online that may help them feel more self-confident, providing facts of successful mentorship connections on the platform, or tools that may reduce the risk of bias around gender, linguistic, or ethnic stereotypes. Last, although this study only tested one type of online mentoring relationship, observational or experimental tests may be used to help see if other types of online mentoring platforms or mentoring programs are impacted with similar interventions.

This study contributes to a broader understanding of how organizations that offer digital mentorship opportunities (Nambisan, 2017; Schou et al., 2021) for entrepreneurs can be more effective. These tools may also be useful in policy and practice to help narrow down those emerging entrepreneurs who are more likely to seek support and use the resources that are provided to them, a potential cost saver for often resource-deprived accelerators, entrepreneurship programs, local governments, and nonprofit organizations. Requiring entrepreneurs to undertake some basic training before engaging on online platforms may increase the barrier to entry but can ultimately lead to more meaningful connections with mentors. These measures, however, should be undertaken carefully to ensure that entrepreneurs from traditionally under-served communities are not left behind. Future research should seek to fine tune these and other interventions to help support motivated entrepreneurs find the resources they need.

## Appendix 2 Description of treatments

### Treatment 1: Video Script for Vicarious Learning

Treatment 1 is a video describing a vivid example of a mentoring relationship is provided below. The video was presented by a member of the platform provider team, and professionally produced. The example description is provided below, and a screenshot of the video in Fig. 1.

### Treatment 2: Enactive Mastery Script for Personalized Chatbot

Treatment 2 is a personalized chatbot that pops up on the screen once an entrepreneur registers for the sight, shows them a sample message to reach out to a mentor, and generates a “small win” by simulating a connection with a mentor (played by the staff member).

### Treatment 3: Verbal Persuasion Script for Automated Chatbot

Treatment 3 is an automated chatbot that pops up on the screen when an entrepreneur signs up for the platform, and tells them about the resources available, as well as some general tips to reach out.

## Footnotes

1 Czibor et al., (2019, p. 7) describe field experiments as experiments that “study the relevant population in a natural setting and, crucially, subjects are not aware of being part of an experiment.”.

2 Accordingly, we report intent-to-treat (ITT) results as our primary findings (as per our experimental pre-registration) since these are generally considered to be more policy-relevant (Angrist & Pischke, 2014; Abadie & Cataneo, 2018). The intent-to-treat analysis captures the causal effect of being assigned to treatment and provides a lower bound of the effectiveness of the treatment. It ignores non-compliance, protocol deviations, and anything that happens after randomization (Angrist & Pischke, 2014).

3 The Spanish versions of the interventions were launched in June 2019 due to delays with the translation process.

4 Entrepreneurs signed up in May would therefore have 6 months to establish connections.

5 One entrepreneur described their hesitation as follows “I didn’t know how it was supposed to work – was I supposed to pay them? were they free for a time?… it didn’t make sense initially and that kept me from interacting further.”.

6 As one entrepreneur on the platform explained, “it takes a lot of effort and courage to reach out.”.

7 Interventions based on these mechanisms were pilot tested with smaller groups of entrepreneurs.

8 The video was offered in both English and Spanish based on the entrepreneur’s choice of language on the platform.

9 The staff member was provided with a script which included variations depending on the entrepreneurs’ response. This intervention was delivered by staff members for the experiment due to cost considerations. If proved effective, the platform provider would invest in programming an AI-based “chatbot” or outsource the task to a customer service provider.

10 The data collection cutoff date is November 4, 2019, 3 months after the field experiment is closed.

11 For completeness, we conduct the same analysis with the male subsample. Unlike the female subsample, none of the treatments has a statistically significant effect on initiating conversation or establishing connections. The table is available upon request.

12 While Google Analytics provides information on total unique watch count of a video, it does not allow us to link the unique watch event to a specific user. Therefore, we are not able to conduct the treatment-on-treated analysis for Treatment 1.

13 To further account for the selection biases, we conduct another set of analysis by restricting the control group to those who sent out at least one message. The effect of Enactive Mastery and Verbal Persuasion on initiating conversation (β= 0.317 and β= 0.340, respectively) and establishing connections (β= 0.657 and β= 0.665, respectively) remain statistically significant at 95% level. The table is available upon request.

14 Unfortunately, we were not able to vary these factors in our field experiment, but the large effect size suggests this is an interesting direction for future research.
